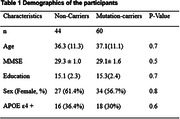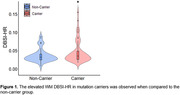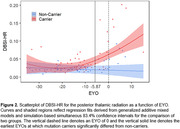# Early Alterations of White Matter Neuroinflammation in Autosomal Dominant Alzheimer's Disease

**DOI:** 10.1002/alz.090382

**Published:** 2025-01-09

**Authors:** Qing Wang, Meng Jiang, Kayle Cohen, Shaney Flores, Sarah J. Keefe, Aakash Patel, Gengsheng Chen, Peter R Millar, Suzanne E. Schindler, Carlos Cruchaga, Brian A. Gordon, John C. Morris, Randall J. Bateman, Eric McDade, Yong Wang, Tammie L.S. Benzinger, Dominantly Inherited Alzheimer Network

**Affiliations:** ^1^ Knight Alzheimer Disease Research Center, Saint Louis, MO USA; ^2^ Washington University in St. Louis School of Medicine, St. Louis, MO USA; ^3^ Washington University in St. Louis, St. Louis, MO USA; ^4^ Washington University School of Medicine, St. Louis, MO USA; ^5^ Washington University in St. Louis, School of Medicine, St. Louis, MO USA; ^6^ Knight Alzheimer Disease Research Center, St. Louis, MO USA; ^7^ NeuroGenomics & Informatics Center, Washington University School of Medicine, St. Louis, MO USA; ^8^ Washington University School of Medicine, Saint Louis, MO USA; ^9^ Hope Center for Neurological Disorders, Washington University School of Medicine, St. Louis, MO USA; ^10^ Department of Radiology, Washington University School of Medicine, Saint Louis, MO USA; ^11^ Hope Center for Neurological Disorders, Washington University in St. Louis, St. Louis, MO USA; ^12^ Knight Alzheimer's Disease Research Center, St. Louis, MO USA

## Abstract

**Background:**

Autosomal Dominant Alzheimer's Disease (ADAD) is a rare and early‐onset form of Alzheimer's disease with a familial pattern of inheritance. While the pathological features of ADAD, such as amyloid plaques and neurofibrillary tangles, have been extensively studied, the involvement of white matter (WM) neuroinflammation is not well‐explored. In sporadic AD, the hindered ratio (HR) derived from diffusion basis spectrum imaging (DBSI) has been used to study neuroinflammation in WM. This study aims to investigate the potential role of WM neuroinflammation as an early driver in the progression of ADAD using DBSI.

**Method:**

This study included 104 participants from the Dominantly Inherited Alzheimer Network (DIAN) Observational Study. 60 participants were identified as mutation carriers of pathologic variants in PSEN1, PSEN2, or APP. Estimated years until symptom onset (EYO) were calculated for each participant based on previous methods (1). Participants’ demographics are summarized in Table 1. Diffusion MRI was acquired using a multi‐b value scheme (bmax =2000 s/mm^2^ and 28 directions) (2). The DBSI‐HR was quantified and obtained from the JHU white matter tracts. Non‐parametric Kruskal‐Wallis and Chi‐square tests were employed to compare demographic variables. The DBSI‐HR on each WM tract as a function of EYO was explored using generalized additive mixed models. All statistical tests were conducted in R4.2.2 (R Core Team). Age, sex, education, and APOE ε4 carrier status were adjusted as covariates.

**Result:**

Demographic data are summarized in Table 1. Mutation carriers had an elevated averaged DBSI‐HR for total WM tracts compared to non‐carriers (Figure 1). Mutation carriers displayed divergent trends from non‐carriers prior to the expected symptom onset for the posterior thalamic radiation (‐5.87 EYO, Figure 2), tapetum (‐2.9 EYO), and sagittal stratum (‐1.20 EYO).

**Conclusion:**

DBSI‐HR measurements indicate increased WM neuroinflammation in the posterior thalamic radiation, tapetum, and sagittal stratum prior to symptom onset in ADAD. We have recently identified these three WM tracts as having the earliest change in DBSI‐HR in sporadic AD. Together, these findings suggest that WM neuroinflammation occurs prior to onset of AD symptoms.